# Construction of the 4-Azafluorenone Core in a Single Operation and Synthesis of Onychine

**DOI:** 10.1021/acs.joc.4c01298

**Published:** 2024-07-17

**Authors:** Victoria A. Lehman, Yun Ma, Jonathan R. Scheerer

**Affiliations:** Department of Chemistry, College of William & Mary, P.O. Box 8795, Williamsburg, Virginia 23187, United States

## Abstract

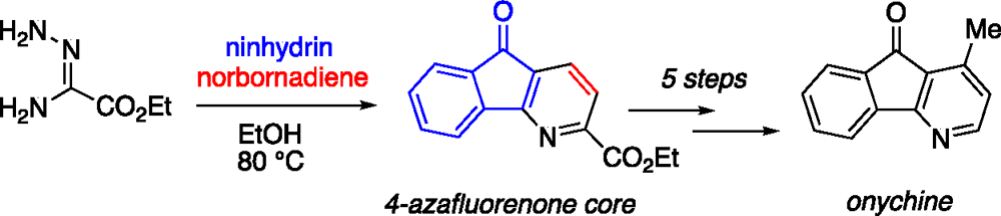

This study describes the synthesis of the 4-azafluorenone core in a single operation using readily available starting materials. Condensation of an amidrazone with ninhydrin intercepts an intermediate 1,2,4-triazine derivative, which engages norbornadiene in a merged [4 + 2]/bis-retro[4 + 2] sequence to deliver the azafluorenone core. The tricyclic core established in this manner was elaborated to onychine, the simplest natural product in the 4-azafluorenone alkaloid family.

4-Azafluorenone natural products have been isolated from plants in the soursop (*Annonacae*) family^1,2^ and other plant species.^3^ Representative alkaloids that possess this tricyclic fused system include onychine (**1**), polyfothine (**2**), and cyathocaline (**3**), and these and related metabolites possess biological activities including antimalarial and cytotoxicity (Figure 1).^4^ Extensive synthetic exploration of azafluorenone derivatives has revealed enhanced antiproliferative characteristics or other properties as compared to the natural products.^5^ As examples, synthetic derivatives **4** and **5** offer improved anticancer activity as compared to the natural azafluorenone variants and are lead compounds for development and potential clinical application.^6,7^ Interest in the 4-azafluorenone core has not been restricted to medicinal chemists; the fundamental photophysical properties have also attracted significant attention.^8,9^ With improved aqueous solubility compared to the parent carbocyclic fluorenone core, azafluorenones have been studied and advanced as fluorescent probes, and in other light-emitting and imaging applications.^10−13^

**Figure 1:**
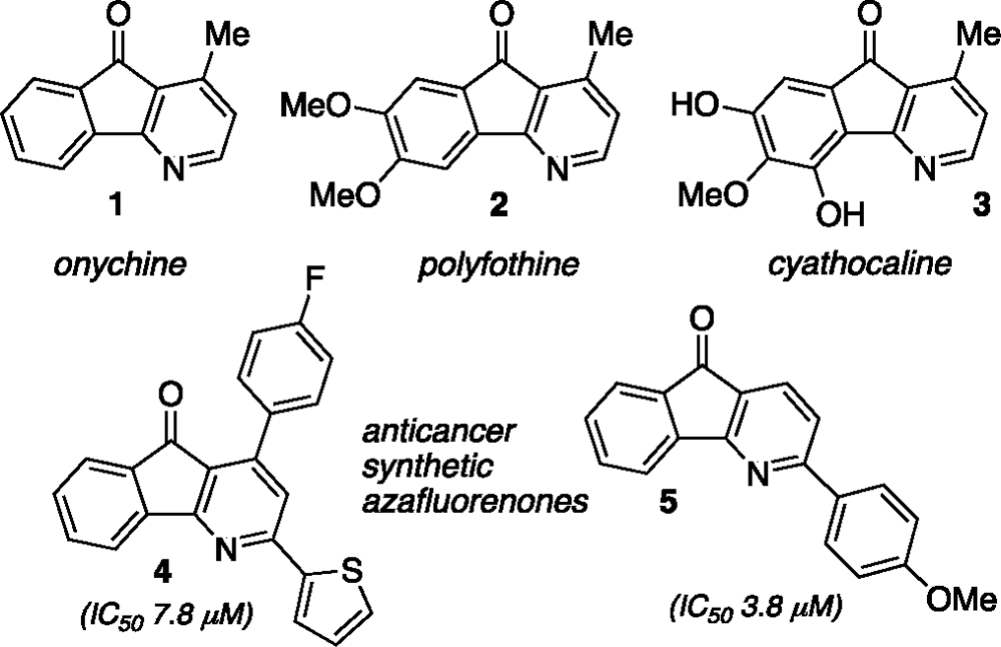
Representative 4-azafluorenone natural products and synthetic derivatives.

Given the broad interest in 4-azafluorenones, several methods for the construction of the nitrogen-containing tricyclic fused system have been reported. If we narrow our focus to onychine (**1**), the simplest and earliest isolated member of the azafluorenone family, we may view the natural product as an instructive model that has been a proving ground of distinct synthetic approaches to the core structure. Synthetic efforts directed toward **1** and related natural products have been recently reviewed.^4^

Our interest in azafluorenes and azafluorenones, as well as in the *de novo* synthesis of pyridine structures, primed us toward a retrosynthesis that identified the substructure of ninhydrin (**8**) embedded within the target molecule (Scheme 1: Retrosynthesis).^14,15^ Given the long and rich history of ninhydrin^16^ derivatives, and the commodity status of the parent ninhydrin (**8**), we envisioned that a synthesis proceeding from this starting material would be direct and relatively versatile for incorporation of substituents on the pyridine nucleus. In this way, condensation of **8** with an amidrazone resembling **9** could lead to an intermediate 1,2,4-triazine **7**, which would enable inverse-electron-demand Diels–Alder (IEDDA) and tandem cycloreversion and rearomatization to deliver the azafluorenone core.^17^ We anticipated that 2π-alkyne equivalent reaction components such as enamines, represented by structures **10a** and **10b**, would not only enable rapid construction of diverse 4-azafluorenone derivatives, but the complementary electronic features of **10a** and **10b** would allow us to modulate the regioselection in the cycloaddition event and permit isolation of **6** with favorable isomeric purity. Although 6-keto-1,2,4-triazines have been prepared in similar fashion, to the best of our knowledge, these precursors have not been explored in tandem [4 + 2]/retro[4 + 2] sequences.^18,19^ Thus, we also saw an opportunity to enter new chemical space quickly and probe the reactivity of a tricyclic triazine resembling **7**.

**Scheme 1:**
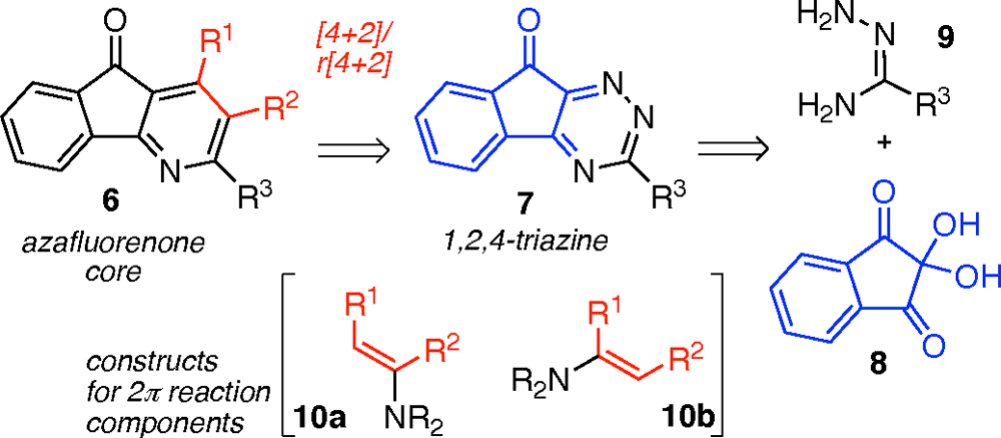
Retrosynthesis of 4-Azafluorenone Core to Ninhydrin Starting Material

In order to evaluate the synthetic viability of the proposed sequence, we selected ethyl oxalamidrazone (**11**) with which to begin our studies (Scheme 2). We chose this amidrazone for several reasons: (1) **11** is well-known, reasonably stable, and benefits from a detailed *Organic Synthesis* procedure for preparation.^20^ (2) We reasoned that the electron-withdrawing ester function would lower the reaction threshold for the IEDDA on the derived triazine **12**. Lastly (3), once the azafluorenone was prepared, the ester could serve as a handle to introduce other functionalities^21−24^ or be resected^25^ to prepare 2-protio azafluorenone structures such as **1**–**3**.

**Scheme 2:**
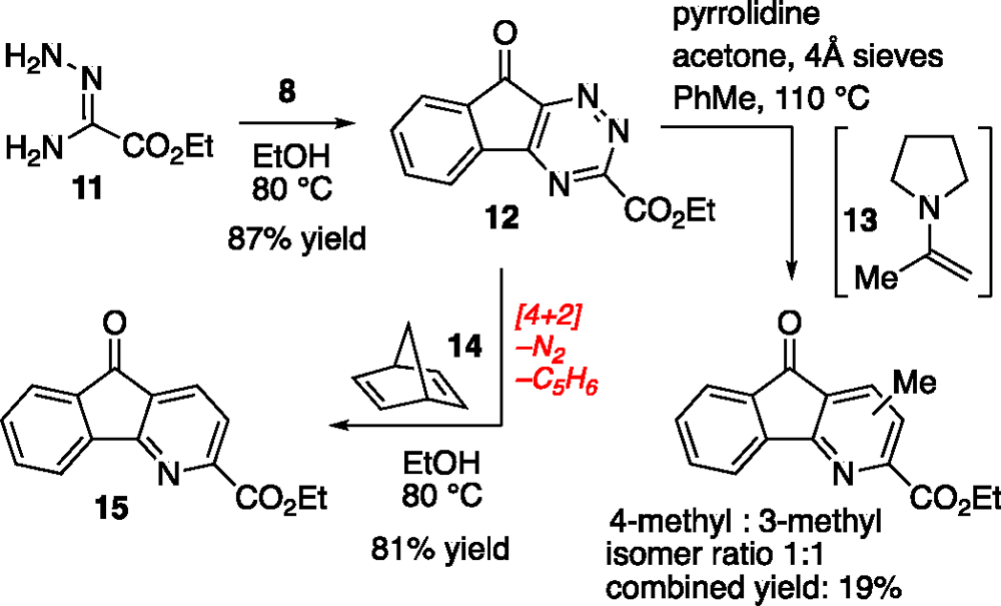
Synthesis of 1,2,4-Triazine and 4-Azafluorenone Core

Synthesis of triazine **12** proceeded efficiently (87% yield) through condensation of amidrazone **11** and ninhydrin (**8**) (Scheme 2). Given the routine use of enamines in IEDDA reactions with 1,2,4-triazines, our preliminary exploration likewise employed enamines as 2π reaction components.^17^ Unfortunately, our results with either *in situ* prepared or preformed enamines, enamides, or enol ethers^26^ derived from acetone or propanal were unimpressive. This exploration of electron-rich alkyne-equivalent heterosubstituted dienophiles failed to offer acceptable yields or regioselectivity. Our best result, using enamine **13** generated *in situ* according to the conditions reported by Taylor and co-workers^27,28^ gave a 19% yield of an inseparable mixture of isomeric azafluorenones (1:1 isomer ratio). The starting triazine **12** was not returned from these reactions and the mass balance was heterogeneous in constitution and other products from the mixture could not be clearly identified. We suspect that the 6-keto functionality in triazine **12** frustrates the desired cycloaddition chemistry with enamine-type dienophiles, leading to poor cycloaddition reaction efficiency.

Given the disappointing results with enamines, we were pleased to discover that a non-nucleophilic alkyne-equivalent 2π reaction component, norbornadiene (**14**), was competent in reaction with triazine **12** and gave azafluorenone **15** in 87% yield on heating in ethanol overnight (80 °C, 16 h). Because both the condensation leading to triazine **12** and the pericyclic cascade to azafluorenone **15** (cycloaddition, and sequential extrusion of N_2_ and cyclopentadiene) are performed in ethanol, we were able to telescope these reactions into a single operation. In practice, we found it most expedient to prepare azafluorenone **15** in this manner, using a single reaction vessel as illustrated in Scheme 3.

**Scheme 3:**
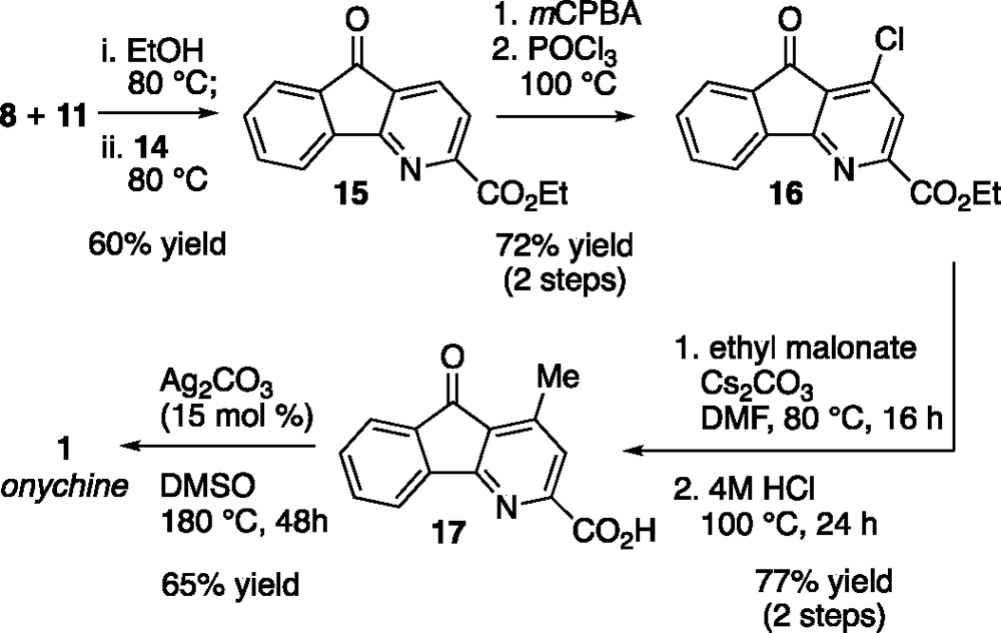
One Step Construction of 4-Azafluorenone Core and Elaboration to Onychine

The use of norbornadiene (**14**) was enabling and provided facile access to the 4-azafluorenone core **15**. With this structure in hand, we turned our attention to functionalization of the pyridine nucleus in **15**. A variety of strategies were envisioned (*e.g*., pyridinium activation,^29−31^ directed metalation,^32,33^ Minisci reaction^34^) and some strategies are partly supported by precedent on similar structures. We found that the hindered 2,6-bis-substituted pyridine nucleus in **15** precluded some chemistry, but the ester at C2 could serve as an effective blocking group that permitted regioselective functionalization at C4 using classic methodology enabled by pyridine *N*-oxide chemistry. To this end, the derived *N*-oxide of azafluorenone **15** was prepared (providing intermediate **S1**) and decomposed with POCl_3_ to give the 4-chloroazafluorenone **16** (2 steps, 60% yield). Nucleophilic substitution of malonate (Cs_2_CO_3_, DMF, 80 °C, 85% yield) on **16** (giving intermediate **S2**) preceded acid-promoted hydrolysis of all ester functional groups and concomitant decarboxylation gave the 4-methyl-2-carboxy azafluorenone **17** (91% yield). Excision of the carboxylic acid functionality at the pyridine C2 position in **17** was performed under silver catalysis (15 mol % Ag_2_CO_3_, DMSO, 180 °C).^25^ Under these reaction conditions, which are modified slightly from the literature, the azafluorenone natural product onychine (**1**) was cleanly obtained. Spectroscopic data for our synthetic material **1** is in agreement with data from other syntheses.^4^

In conclusion, we have discovered a direct method to prepare the core 4-azafluorenone structure in a single reaction vessel from readily available starting materials. The condensation operations with amidrazone and ninhydrin intercepted an intermediate 1,2,4-triazine and also proved compatible with the pericyclic reaction steps. Use of norbornadiene as the alkyne-equivalent 2π reaction component with the intermediate 1,2,4-triazine was integral to a successful cycloaddition. The ensuing cycloreversion steps (extrusion of N_2_ and cyclopentadiene) proceeded in tandem fashion without isolation of intermediate adducts. The 4-azafluorenone product obtained in this fashion was elaborated to onychine, a model natural product in this family. The “one pot” construction of the azafluorenone core disclosed in this note is efficient and complementary to existing synthetic strategies. We are optimiztic that the efficient construction of the 4-azafluorenone core central to this work will enable downstream advances in both the medicinal and material applications of this privileged scaffold.

## Experimental Section

### General Experimental Considerations

All reactions were carried out under an atmosphere of nitrogen in flame-dried or oven-dried glassware with magnetic stirring unless otherwise indicated. Dichloromethane was distilled from CaH_2_ prior to use. All other reagents were used as received unless otherwise noted. Flash column chromatography was performed using P60 silica gel (230–400 mesh). Analytical thin layer chromatography was performed on SiliCycle 60 Å glass plates. Visualization was accomplished with UV light, anisaldehyde, ceric ammonium molybdate, potassium permanganate, or ninhydrin, followed by heating. Film infrared spectra were recorded using a Digilab FTS 7000 FTIR spectrophotometer. ^1^H NMR spectra were recorded on a Varian Mercury 400 (400 MHz) spectrometer and are reported in ppm using solvent as an internal standard (CHCl_3_ at 7.26 ppm) or tetramethylsilane (0.00 ppm). Proton-decoupled ^13^C NMR spectra were recorded on a 100 MHz spectrometer and are reported in ppm using solvent as an internal standard (CHCl_3_ at 77.0 ppm or DMSO at 39.5 ppm). All compounds were judged to be homogeneous (>95% purity) by ^1^H and ^13^C NMR spectroscopy unless otherwise noted. Mass spectra data analysis was obtained through positive electrospray ionization (w/NaCl) on a Bruker 12 T APEX–Qe FTICR-MS with an Apollo II ion source using an ICR (ion cyclotron resonance) ion trap mass analyzer.
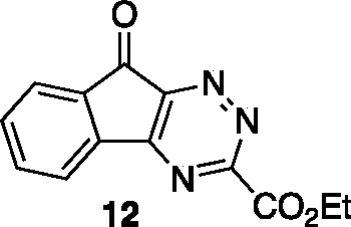


### Ethyl 9-Oxo-9*H*-indeno[1,2-*e*][1,2,4]triazine-3-carboxylate (**12**)

A dry flask was charged with ethyl oxalamidrazonate^20^ (**11**) (6.40 g, 48.7 mmol) and ninhydrin (8.5 g, 48.7 mmol) and dissolved in EtOH (150 mL). The flask was fitted with a condenser, flushed with N_2_, and heated to reflux using an aluminum block. After heating for 1 h, the reaction mixture was cooled to 23 °C and concentrated *in vacuo.* The resulting mixture was purified by flash column chromatography on silica gel (gradient elution: 0% EtOAc to 20% EtOAc in hexanes and an isocratic additive of 50% CH_2_Cl_2_) to afford triazine **12** (10.9 g, 41.8 mmol, 87% yield) as a yellow amorphous solid. TLC (15% EtOAc, 35% hexane, 50% CH_2_Cl_2_), R_*f*_. 0.33 (UV/KMnO_4_); IR (film) 1728, 1558, 1242, 756 cm^–1^; ^1^H NMR (400 MHz, CDCl_3_) 8.06–8.00 (m, 3H), 7.76 (d, *J* = 7.4 Hz, 1H), 7.65 (dd, *J*_1_ = 8.4 Hz, *J*_2_ = 0.93 Hz, 1H), 7.48 (dd, *J*_1_ = 8.4 Hz, *J*_2_ = 0.93 Hz, 1H), 4.52 (q, *J* = 7.1 Hz, 2H), 1.48 (t, *J* = 7.1 Hz, 3H); ^13^C {^1^H}NMR (100 MHz, CDCl_3_) δ 186.1, 163.7, 162.1, 157.0, 153.1, 137.7, 137.3, 135.7, 135.4, 125.5, 125.0, 63.5, 14.0; HRMS (ESI) *m*/*z*: [M + Na]^+^ Calcd for C_13_H_9_N_3_O_3_Na^+^ 278.0536; Found 278.0536.
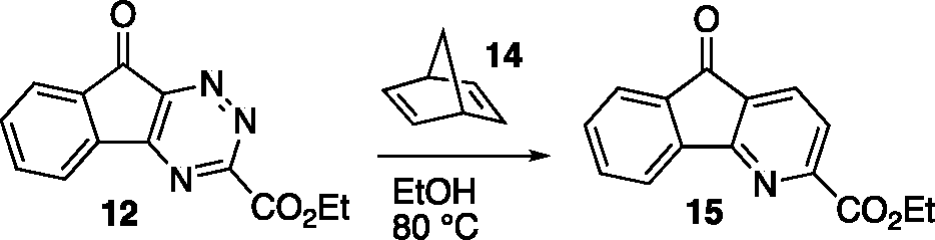


### Ethyl 5-Oxo-5*H*-indeno[1,2-*b*]pyridine-2-carboxylate (**15**)

A dry flask was charged with norbornadiene (0.742 g, 2.91 mmol) and **12** (1.48 mL, 14.5 mmol) and dissolved in EtOH (15 mL). The reaction was fitted with a condenser, flushed with N_2_, and heated to reflux using an aluminum block. After heating at reflux for 16 h, the reaction mixture was cooled to 23 °C, and concentrated *in vacuo.* The resulting mixture was purified by flash column chromatography on silica gel (gradient elution: 0% EtOAc to 40% EtOAc in hexanes) to afford **15** (0.595 g, 2.35 mmol, 81% yield) as a yellow powder. Mp: 130–132 °C; TLC (20% EtOAc in hexane), R_*f*_. 0.25 (UV/KMnO_4_); IR (film) 3100, 1717, 1307, 1098, 741 cm^–1^; ^1^H NMR (400 MHz, CDCl_3_) 8.06–8.00 (m, 3H), 7.76 (d, *J* = 7.4 Hz, 1H), 7.65 (dd, *J*_1_ = 8.4 Hz, *J*_2_ = 0.93 Hz, 1H), 7.48 (dd, *J*_1_ = 8.4 Hz, *J*_2_ = 0.93 Hz, 1H), 4.52 (q, *J* = 7.4 Hz, 2H), 1.48 (t, *J* = 7.0 Hz, 3H); ^13^C{1H} NMR (100 MHz, CDCl_3_) δ 190.7, 165.1, 164.5, 151.9, 143.0, 135.6, 135.1, 131.9, 131.5, 130.7, 125.0, 124.3 122.0, 62.3, 14.2; HRMS (ESI) *m*/*z*: [M + Na]^+^ Calcd for C_15_H_11_NO_3_Na^+^ 276.0631; Found 276.0632.
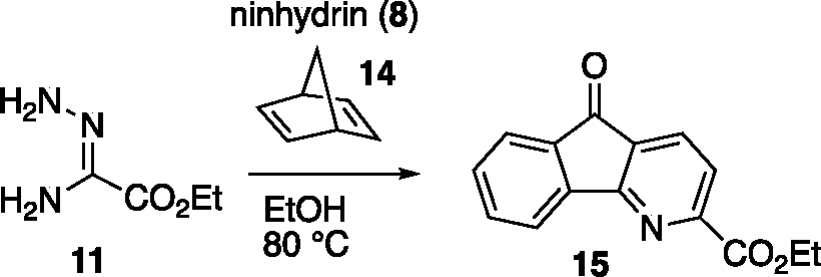


#### “One Pot” Method for the Preparation of Azafluorenone **15.**

A dry flask was charged with ethyl oxalamidrazonate (**11**) (1.93 g, 14.6 mmol) and ninhydrin (**8**) (2.61 g, 14.6 mmol) and dissolved in EtOH (40 mL). The flask was fitted with a reflux condenser, flushed with N_2_, and heated to reflux using an aluminum block. After heating at reflux for 1 h, to the reaction was added norbornadiene (**14**) (6.7 mL, 73.0 mmol). Reaction heating was continued for an additional 16 h, after which time the reaction was cooled to 23 °C and concentrated *in vacuo.* The resulting mixture was purified by flash column chromatography as indicated above to afford **15** (2.23 g, 8.8 mmol, 60% yield) as a yellow powder. Spectral data for **15** is described above.
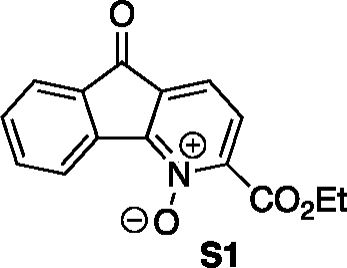


### 2-(Ethoxycarbonyl)-5-oxo-5*H*-indeno[1,2-*b*]pyridine 1-Oxide (**S1**)

A flask was charged **15** (0.37 g, 1.46 mmol), dissolved in CH_2_Cl_2_ (8 mL) and *m*-CBPA (77% by weight, 0.76 g, 3.38 mmol) was added. The reaction was stirred for 20 h at 23 °C. The reaction was diluted with NaHCO_3_ (10 mL) and the aqueous layer was extracted with CH_2_Cl_2_ (3 × 15 mL). The combined organic layers were dried with MgSO_4_, filtered, and concentrated *in vacuo*. The resulting mixture was purified by flash column chromatography on silica gel (gradient elution: 0% EtOAc to 80% EtOAc in hexanes) to afford the desired pyridine *N*-oxide (0.99 g, 3.72 mmol, 85% yield) as an amber solid. Mp: 140–145 °C; TLC (40% EtOAc in hexane), R_*f*_. 0.38 (UV/KMnO_4_); IR (film) 2985, 1720, 1604, 1280, 1103, 740 cm^–1^; ^1^H NMR (400 MHz, CDCl_3_) 8.64 (d, *J* = 7.2 Hz, 1H), 7.79 (d, *J* = 7.6 Hz, 1H), 7.66 (td, *J*_1_ = 7.6 Hz, *J*_2_ = 0.93 Hz, 1H), 7.52–7.50 (m, 3H), 4.53 (q, *J* = 7.2 Hz, 2H), 1.46 (t, *J* = 7.2 Hz, 3H); ^13^C{1H} NMR (100 MHz, CDCl_3_) δ 188.4, 161.0, 147.2, 137.6, 135.9, 133.1, 132.8, 131.7, 126.0, 125.9, 125.9, 124.6, 119.5, 63.0, 14.1; HRMS (ESI) *m*/*z*: [M + Na]^+^ Calcd for C_15_H_11_NO_4_Na^+^ 292.0580; Found 292.0582.
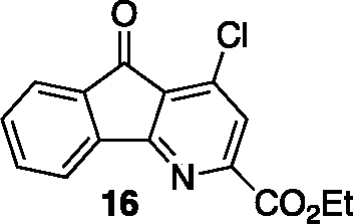


### Ethyl 4-Chloro-5-oxo-5*H*-indeno[1,2-*b*]pyridine-2-carboxylate (**16**)

A flask was charged with 2-(ethoxycarbonyl)-5-oxo-5*H*-indeno[1,2-*b*]pyridine 1-oxide (2.93 g, 11.0 mmol) and fitted with a condenser. To the reaction was added POCl_3_ (30 mL) and the reaction vessel was heated using an aluminum block set to 100 °C. After heating for 3 h, the reaction was cooled to 23 °C and poured onto a mixture of ice (ca. 100 g) and CH_2_Cl_2_ (50 mL). The pH of the aqueous layer was adjusted to 8 by adding solid K_2_CO_3_. The organic layer was removed and the aqueous layer was extracted with CH_2_Cl_2_ (5 × 10 mL). The combined organic layers were dried with Na_2_SO_4_, filtered, and concentrated *in vacuo*. The resulting mixture was purified by flash column chromatography on silica gel (gradient elution: 0% EtOAc to 40% EtOAc in hexanes) to afford chloropyridine **16** (2.67 g, 9.28 mmol, 85% yield) as a yellow solid. Mp: 158–162 °C; TLC (20% EtOAc in hexane), R*f*: 0.37 (UV/KnMO_4_); IR (film) 2970, 2322, 1720, 1566, 750 cm^–1^; ^1^H NMR (400 MHz, CDCl_3_) 8.05 (d, *J* = 7.2 Hz, 1H), 7.95 (s, 1H), 7.79 (d, *J* = 7.6 Hz, 1H), 7.67 (t, *J* = 7.5 Hz, 1H), 7.52 (t, *J* = 7.6 Hz, 1H), 4.52 (q, *J* = 7.1 Hz, 2H), 1.48 (t, *J* = 7.1 Hz, 3H); ^13^C{^1^H} NMR (100 MHz, CDCl_3_) δ 187.8, 166.4, 163.5, 152.1, 142.3, 141.4, 135.6, 134.9, 131.9, 126.7, 126.5, 124.3, 122.2, 62.6, 14.1; HRMS (ESI) *m*/*z*: [M + Na]^+^ Calcd for C_15_H_10_NO_3_ClNa^+^ 310.0241; Found 310.0243.
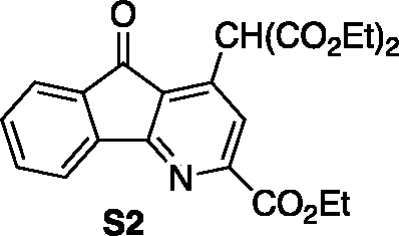


### Diethyl 2-(2-(Ethoxycarbonyl)-5-oxo-5*H*-indeno[1,2-*b*]pyridin-4-yl)malonate (**S2**)

A dry vial was charged with chloropyridine **16** (0.10 g, 0.35 mmol) and dissolved in DMF (1.7 mL). Diethyl malonate (0.065 mL, 0.42 mmol) and Cs_2_CO_3_ (0.227 g, 0.70 mmol) were added sequentially. The vial was sealed with a Teflon cap and heated using an aluminum block set to 80 °C. After stirring for 16 h at 80 °C, the reaction was cooled to 23 °C, diluted with H_2_O, and acidified with 1 M HCl until the pH of the aqueous layer was 4. The mixture was extracted with CH_2_Cl_2_ (3 × 5 mL), and the combined organic layers were washed with brine, dried over Na_2_SO_4_, filtered, and concentrated *in vacuo*. The resulting mixture was purified by flash column chromatography on silica gel (gradient elution: 0% EtOAc to 40% EtOAc in hexanes) to afford the desired product (Diethyl 2-(2-(ethoxycarbonyl)-5-oxo-5*H*-indeno[1,2-*b*]pyridin-4-yl)malonate) (0.12 g, 0.297 mmol, 85% yield) as a yellow solid. Mp: 155–160 °C; TLC (30% EtOAc in hexane), R*f*: 0.52 (UV/KMnO_4_); IR (film) 2984, 1748, 1732, 1717, 1209, 1155, 1020, 733 cm^–1^; ^1^H NMR (400 MHz, CDCl_3_) 8.12 (s, 1H), 8.06 (d, *J* = 7.6 Hz, 1H), 7.74 (d, *J* = 7.6 Hz, 1H), 7.65 (t, *J* = 7.4 Hz, 1H), 7.48 (t, *J* = 7.6 Hz, 1H), 5.99 (s, 1H), 4.91 (q, *J* = 7.2 Hz, 2H), 4.33–4.24 (m, 4H), 1.47 (t, *J* = 7.2 Hz, 3H), 1.30 (t, *J* = 9.2 Hz, 6H); ^13^C{^1^H} NMR (100 MHz, CDCl_3_) δ 191.4, 166.3, 165.2, 164.2, 142.4, 141.4, 135.7, 135.0, 131.5, 127.6, 125.5, 124.2, 122.0, 62.4, 62.3, 51.0, 14.2, 14.1, 13.9; HRMS (ESI) *m*/*z*: [M + Na]^+^ Calcd for C_22_H_21_NO_7_Na^+^ 434.1202; Found 434.1211.
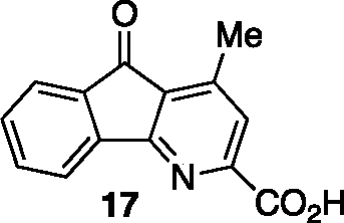


### 4-Methyl-5-oxo-5*H*-indeno[1,2-*b*]pyridine-2-carboxylic Acid (**17**)

To a vial was added pyridine diethyl 2-(2-(ethoxycarbonyl)-5-oxo-5*H*-indeno[1,2-*b*]pyridin-4-yl)malonate (0.025 g, 0.06 mmol) and 4 M aqueous HCl (0.5 mL). The vial was sealed with a Teflon cap and heated using an aluminum block set to 100 °C. After heating for 16 h, the vial was cooled to 23 °C, diluted with water (5 mL) and the pH was adjusted to 4 using 1 M NaOH and extracted with EtOAc (5 × 5 mL). The combined organic layers were dried over Na_2_SO_4_, filtered, and concentrated *in vacuo* to afford **17** (0.013 g, 0.054 mmol, 91% yield) as a yellow solid that was used without further purification. Mp: 150–155 °C; TLC (10% MeOH in CHCl_3_), R*f*. 0.33 (UV/KMnO_4_); IR (film) 1981, 1743, 1718, 1703, 1574, 1415, 1311, 1294, 751 cm^–1^; ^1^H NMR (400 MHz, CDCl_3_) 7.96 (s, 1H), 7.90 (d, *J* = 7.6 Hz, 1H), 7.78 (d, *J* = 7.2 Hz, 1H), 7.67 (t, *J = 7.6 Hz*, 1H), 7.5 (td, *J*_1_ = 7.5 Hz, *J*_2_ = 0.93 Hz, 1H), 2.76 (s, 3H); ^13^C{^1^H} NMR (100 MHz, DMSO) δ 191.8, 181.4, 165.7, 164.3, 151.6, 148.2, 142.1, 135.9, 134.8, 131.7, 127.4, 123.8, 121.1, 16.8; HRMS (ESI) *m*/*z*: [M + Na]^+^ Calcd for C_14_H_9_NO_3_Na^+^ 262.0475; Found 262.0475.
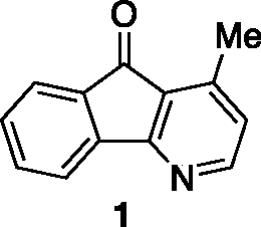


### Onychine (**1**)

The following silver-catalyzed protodecarboxylation is based on related decarboxylation of pyridine-2-carboxylic acids.^25^ To a vial was added azafluorenone **17** (0.050 g, 0.21 mmol), Ag_2_CO_3_ (0.008 g, 0.032 mmol), and DMSO (0.420 mL). The vial was sealed with a Teflon cap and heated using an aluminum block set to 170 °C. After 48 h, the reaction vessel was cooled to rt, diluted with sat. aqueous NaHCO_3_ (3 mL) and extracted with CH_2_Cl_2_ (5 × 3 mL). The organic layers were combined, washed with brine (2 × 2 mL), dried with Na_2_SO_4_, filtered and concentrated *in vacuo*. The resulting mixture was purified by flash column chromatography on silica gel (gradient elution: 0% EtOAc to 20% EtOAc in hexanes) to afford **1** (0.030 g, 0.137 mmol, 65% yield) as a light yellow solid. The spectral data obtained for **1** (^1^H and ^13^C NMR tabulated below) are in agreement with that reported in literature.^4^^1^H NMR (400 MHz, CDCl_3_) 8.43 (d, *J* = 5.2 Hz, 1H), 7.84 (d, *J* = 7.6 Hz, 1H), 7.70 (d, *J* = 7.6 Hz, 1H), 7.59 (t, *J* = 7.2 Hz, 1H), 7.43 (t, *J* = 7.6 Hz, 1H), 6.97 (d, *J* = 5.6 Hz, 1H), 2.64 (s, 3H); ^13^C{^1^H} NMR (100 MHz, CDCl_3_) δ 193.3, 165.3, 152.9, 147.6, 143.1, 135.1, 135.0, 130.8, 126.0, 125.9, 123.7, 120.8, 17.3.

## Data Availability

The data underlying this study are available in the published article and the Supporting Information.

## Supporting Information Available

^1^H and ^13^C NMR spectra for all new compounds (PDF)

## References

[ref1] De Almeida M. E. L.; Braz F R.; von Bülow V.; Gottlieb O. R.; Maia J. G. S. Onychine, an Alkaloid from Onychopetalum Amazonicum. Phytochemistry 1976, 15, 1186–1187. 10.1016/0031-9422(76)85134-5.

[ref2] Achenbach H.; Schwinn A. Constituents of Tropical Medicinal Plants, LXIII: Synthesis of 2 Methoxyonychine Alkaloids Structure Revision of Oxylopidine. Arch. Pharm. (Weinheim) 1994, 327, 755–762. 10.1002/ardp.19943271202.

[ref3] Prachayasittikul S.; Manam P.; Chinworrungsee M.; Isarankura-Na-ayudhya C.; Ruchirawat S.; Prachayasittikul V. Bioactive Azafluorenone Alkaloids from Polyalthia Debilis (Pierre) Finet & Gagnep. Molecules 2009, 14, 4414–4424. 10.3390/molecules14114414. 19924075 PMC6255371

[ref4] Jourjine I. A. P.; Bracher F. Collective Total Synthesis of 4-Azafluorenone Alkaloids. Eur. J. Org. Chem.. 2023, 26 ( (28), ). 10.1002/ejoc.202300399.

[ref5] Gomes C. R. B.; de Souza M. V. N.; Facchinetti V. A Review on Onychine and Its Analogs: Synthesis and Biological Activity. Curr. Org. Synth. 2020, 17, 3–22. 10.2174/1570179417666191218112842. 32103713

[ref6] Marquise N.; Chevallier F.; Nassar E.; Frédérich M.; Ledoux A.; Halauko Y. S.; Ivashkevich O. A.; Matulis V. E.; Roisnel T.; Dorcet V.; Mongin F. Substituted Azafluorenones: Access from Dihalogeno Diaryl Ketones by Palladium-Catalyzed Auto-Tandem Processes and Evaluation of Their Antibacterial, Antifungal, Antimalarial and Antiproliferative Activities. Tetrahedron 2016, 72, 825–836. 10.1016/j.tet.2015.12.050.

[ref7] Gul H. I.; Tugrak M.; Gul M.; Sakagami H.; Umemura N.; Anil B. Synthesis and Cytotoxicities of New Azafluorenones with Apoptotic Mechanism of Action and Cell Cycle Analysis. Anticancer Agents Med. Chem. 2019, 18, 1770–1778. 10.2174/1871520618666180525085445. 29793413

[ref8] Biczók L. Photophysical Properties of 3-Azafluorenone. React. Kinet. Catal. Lett. 1997, 61, 57–62. 10.1007/BF02477513.

[ref9] Biczók L.; Cser A.; Nagy K. Substituent and Solvent Effects on the Photophysical Properties of 3-Azafluorenone Derivatives. J. Photochem. Photobiol. Chem. 2001, 146 (1–2), 59–62. 10.1016/S1010-6030(01)00597-4.

[ref10] Ye F.; Tran C.; Jullien L.; Le Saux T.; Haddad M.; Michelet V.; Ratovelomanana-Vidal V. Synthesis of Fluorescent Azafluorenones and Derivatives via a Ruthenium-Catalyzed [2 + 2 + 2] Cycloaddition. Org. Lett. 2018, 20, 4950–4953. 10.1021/acs.orglett.8b02085. 30070483

[ref11] Gao M.; Su H.; Lin Y.; Ling X.; Li S.; Qin A.; Tang B. Z. Photoactivatable Aggregation-Induced Emission Probes for Lipid Droplets-Specific Live Cell Imaging. Chem. Sci. 2017, 8, 1763–1768. 10.1039/C6SC04842K. 29780451 PMC5933432

[ref12] Sharma A.; Umar S.; Kar P.; Singh K.; Sachdev M.; Goel A. A New Type of Biocompatible Fluorescent Probe AFN for Fixed and Live Cell Imaging of Intracellular Lipid Droplets. Analyst 2016, 141, 137–143. 10.1039/C5AN01623A. 26528832

[ref13] Liu X.-Y.; Zhang Y.-J.; Fei X.; Ran Q.; Fung M.-K.; Fan J. Diazaspirocycles: Novel Platforms for Efficient Phosphorescent Organic Light-Emitting Diodes. J. Mater. Chem. C 2019, 7, 1370–1378. 10.1039/C8TC05462B.

[ref14] Angello N. H.; Wiley R. E.; Abelt C. J.; Scheerer J. R. Synthesis and Spectrophotometric Analysis of 1-Azafluorenone Derivatives. Molecules 2020, 25, 3358–3366. 10.3390/molecules25153358. 32722081 PMC7436005

[ref15] Carrillo Vallejo N. A.; Scheerer J. R. Application of 1,4-Oxazinone Precursors to the Construction of Pyridine Derivatives by Tandem Intermolecular Cycloaddition/Cycloreversion. J. Org. Chem. 2021, 86, 5863–5869. 10.1021/acs.joc.1c00288. 33797249 PMC8394597

[ref16] Teeters W. O.; Shriner R. L. A New Preparation of Ninhydrin. J. Am. Chem. Soc. 1933, 55, 3026–3028. 10.1021/ja01334a072.

[ref17] Zhang F.-G.; Chen Z.; Tang X.; Ma J.-A. Triazines: Syntheses and Inverse Electron-Demand Diels-Alder Reactions. Chem. Rev. 2021, 121, 14555–14593. 10.1021/acs.chemrev.1c00611. 34586777

[ref18] Wu X.; Li X.; Li Z.; Yu Y.; You Q.; Zhang X. Discovery of Nonquinone Substrates for NAD(P)H: Quinone Oxidoreductase 1 (NQO1) as Effective Intracellular ROS Generators for the Treatment of Drug-Resistant Non-Small-Cell Lung Cancer. J. Med. Chem. 2018, 61, 11280–11297. 10.1021/acs.jmedchem.8b01424. 30508483

[ref19] Ried W.; Schomann P. Reaktionen von Amidrazonen Mit Vicinalen Triketonen. Justus Liebigs Ann. Chem. 1968, 714, 128–139. 10.1002/jlac.19687140113.

[ref20] Boger D. L.; Panek J. S.; Yasuda M. Preparation and Inverse-Electron-Demand Diels-Alder Reaction of an Electron-Deficient Heterocyclic Azadiene: Triethyl 1,2,4-Triazine-3,5,6-Tricarboxylate and 2,3,6-Tricarboethoxypyridine. Org. Synth, 1988, 66, 142 10.15227/orgsyn.066.0142.

[ref21] Chen T. Q.; Pedersen P. S.; Dow N. W.; Fayad R.; Hauke C. E.; Rosko M. C.; Danilov E. O.; Blakemore D. C.; Dechert-Schmitt A.-M.; Knauber T.; Castellano F. N.; Macmillan D. W. C. A Unified Approach to Decarboxylative Halogenation of (Hetero)Aryl Carboxylic Acids. J. Am. Chem. Soc. 2022, 144, 8296–8305. 10.1021/jacs.2c02392. 35486956 PMC9676088

[ref22] Cornella J.; Larrosa I. Decarboxylative Carbon-Carbon Bond-Forming Transformations of (Hetero)Aromatic Carboxylic Acids. Synthesis 2012, 44, 653–676. 10.1055/s-0031-1289686.

[ref23] Font M.; Quibell J. M.; Perry G. J. P.; Larrosa I. The Use of Carboxylic Acids as Traceless Directing Groups for Regioselective C-H Bond Functionalisation. Chem. Commun. 2017, 53, 5584–5597. 10.1039/C7CC01755C. 28492623

[ref24] Johnston A. J. S.; Ling K. B.; Sale D.; Lebrasseur N.; Larrosa I. Direct Ortho-Arylation of Pyridinecarboxylic Acids: Overcoming the Deactivating Effect of Sp2-Nitrogen. Org. Lett. 2016, 18, 6094–6097. 10.1021/acs.orglett.6b03085. 27934340

[ref25] Lu P.; Sanchez C.; Cornella J.; Larrosa I. Silver-Catalyzed Protodecarboxylation of Heteroaromatic Carboxylic Acids. Org. Lett. 2009, 11, 5710–5713. 10.1021/ol902482p. 19924891

[ref26] Rocha Gonsalves A. M. d.’A.; Pinhoe Melo T. M. V. D.; Gilchrist T. L. Diels-Alder Reactions of 1,2,4-Triazines with Cyclic Vinyl Ethers. Tetrahedron 1993, 49, 5277–5290. 10.1016/S0040-4020(01)82377-2.

[ref27] Bromley W. J.; Gibson M.; Lang S.; Raw S. A.; Whitwood A. C.; Taylor R. J. K. Tandem Inverse Electron Demand Diels-Alder, Retro-Diels-Alder and Intramolecular Diels-Alder Sequences: One-Pot Synthesis of Diaza-Polycycles. Tetrahedron 2007, 63, 6004–6014. 10.1016/j.tet.2007.02.056.

[ref28] Raw S. A.; Taylor R. J. K. Recent Advances in the Chemistry of 1,2,4-Triazines. Advances in Heterocyclic Chemistry 2010, 100, 100 10.1016/S0065-2725(10)10003-8.

[ref29] Obradors C.; List B. Azine Activation via Silylium Catalysis. J. Am. Chem. Soc. 2021, 143, 6817–6822. 10.1021/jacs.1c03257. 33908753 PMC8154516

[ref30] Dolewski R. D.; Hilton M. C.; McNally A. 4-Selective Pyridine Functionalization Reactions via Heterocyclic Phosphonium Salts. Synlett 2018, 29, 8–14. 10.1055/s-0036-1591850.

[ref31] Josephitis C. M.; Nguyen H. M. H.; McNally A. Late-Stage C-H Functionalization of Azines. Chem. Rev. 2023, 123, 7655–7691. 10.1021/acs.chemrev.2c00881. 37134187 PMC10631472

[ref32] Mongin F.; Quéguiner G. Advances in the Directed Metallation of Azines and Diazines (Pyridines, Pyrimidines, Pyrazines, Pyridazines, Quinolines, Benzodiazines and Carbolines). Part 1: Metallation of Pyridines, Quinolines and Carbolines. Tetrahedron 2001, 57, 4059–4090. 10.1016/S0040-4020(01)00100-4.

[ref33] Schlosser M.; Mongin F. Pyridine Elaboration through Organometallic Intermediates: Regiochemical Control and Completeness. Chem. Soc. Rev. 2007, 36, 1161–1172. 10.1039/b706241a. 17576483

[ref34] Choi J.; Laudadio G.; Godineau E.; Baran P. S. Practical and Regioselective Synthesis of C-4-Alkylated Pyridines. J. Am. Chem. Soc. 2021, 143, 11927–11933. 10.1021/jacs.1c05278. 34318659 PMC8721863

