# Supplementary material for: Construction of the 4-Azafluorenone Core in a Single Operation and Synthesis of Onychine
Source: J Org Chem. 2024 Jul 17;89(15):11078–82. doi: 10.1021/acs.joc.4c01298 (PMC11301660; doi:10.1021/acs.joc.4c01298)
Supplement: Supplementary file 1 — jo4c01298_si_001.pdf [file jo4c01298_si_001.pdf]

Supporting Information

Construction of the 4-Azafluorenone Core in a Single Operation and Synthesis of Onychine

Victoria A. Lehman, Yun Ma, Jonathan R. Scheerer*

Department of Chemistry, The College of William & Mary, P.O. Box 8795, Williamsburg, Virginia, 23187.

Supporting Information

^{1}H and ^{13}C NMR Spectra S2–S8

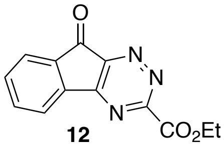

12

^{1}H
400 MHz
$CDCl_3$

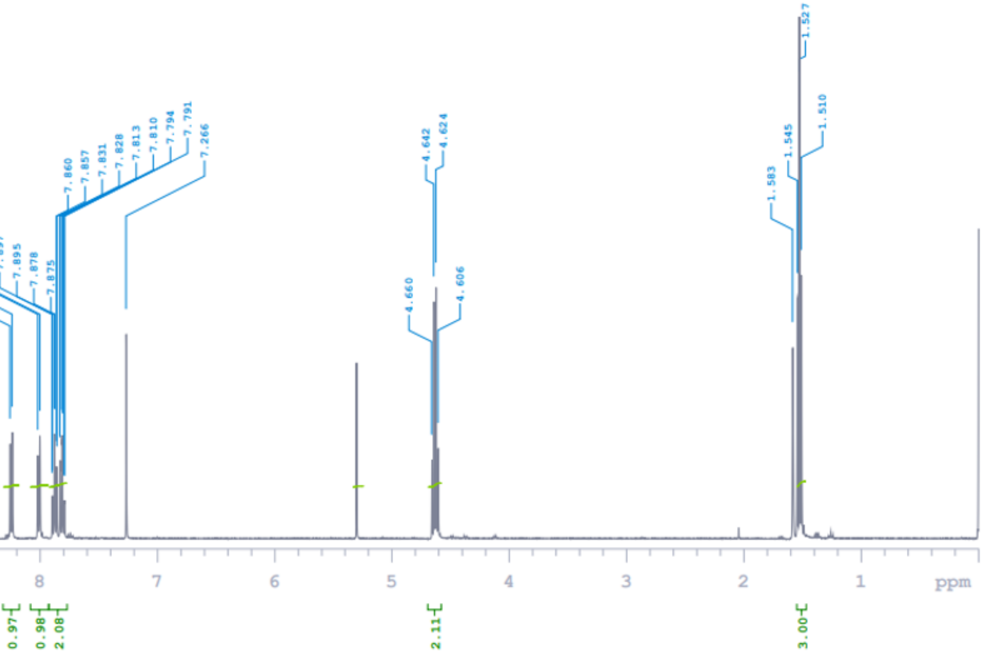

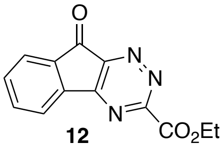

12

$^{13}C\{^{1}H\}$
100 MHz
$CDCl_3$

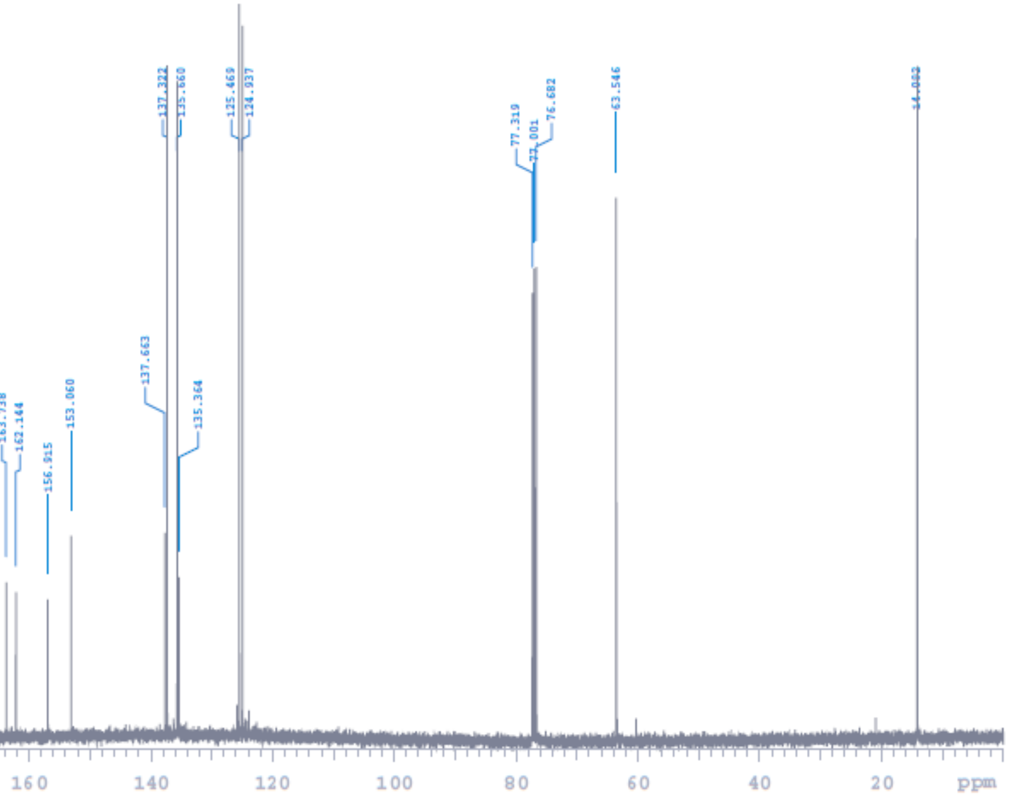

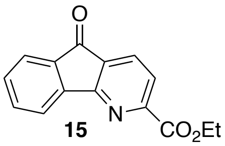

15

^1H
400 MHz
$CDCl_3$

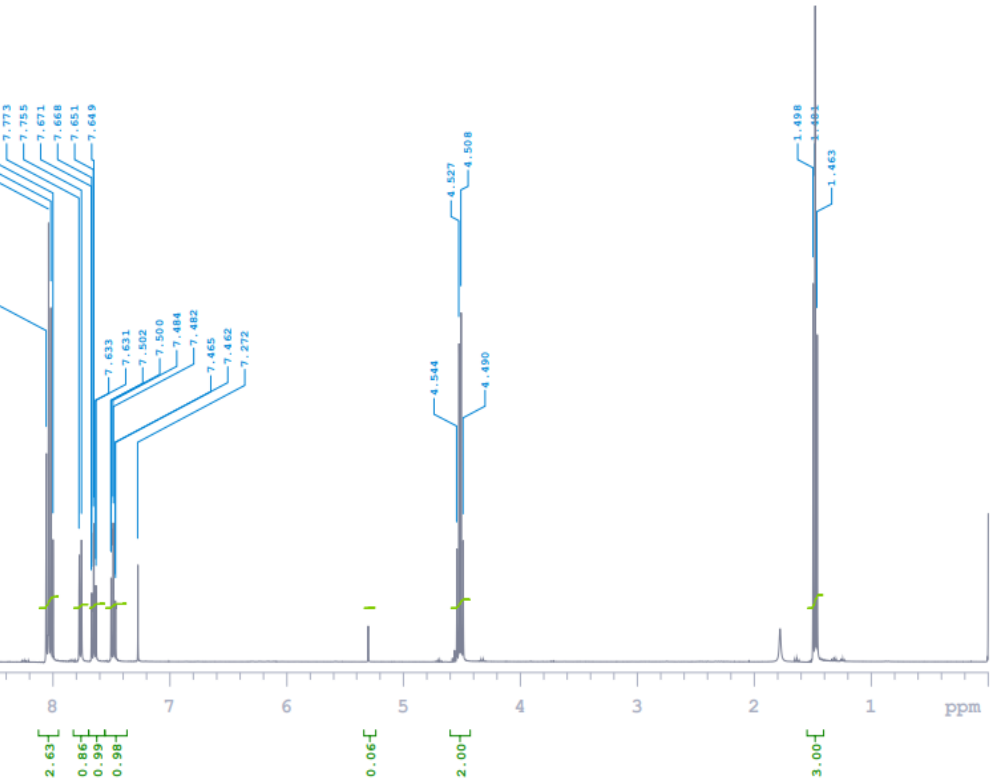

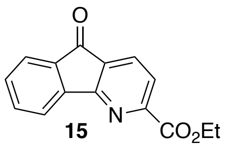

15

$^{13}C\ \{^1H\}$
100 MHz
$CDCl_3$

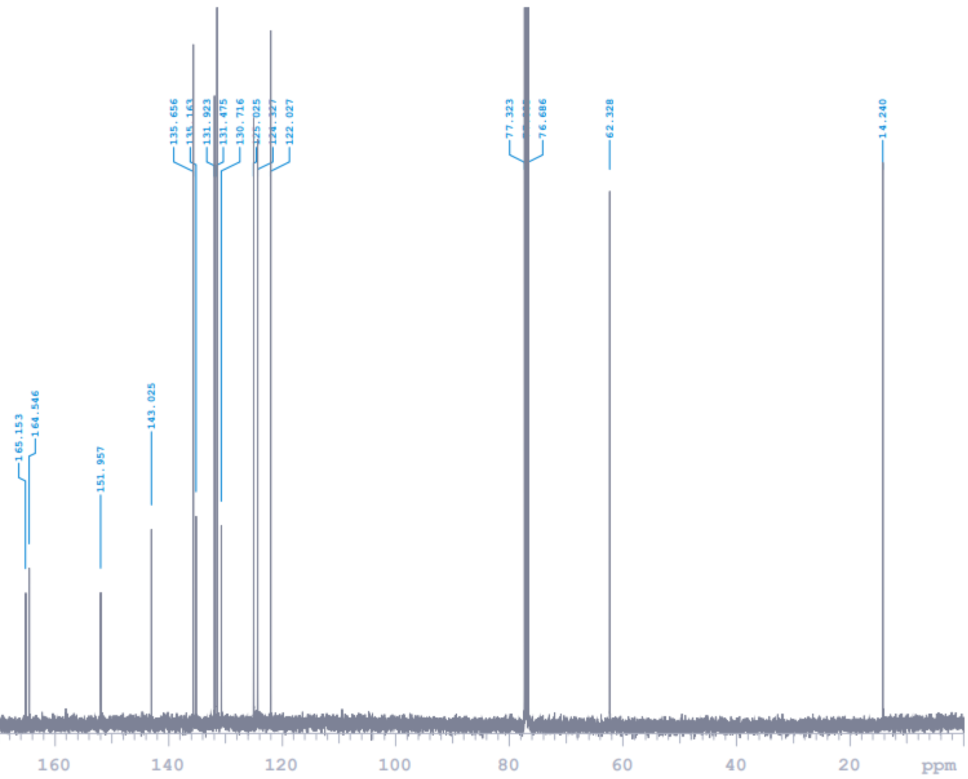

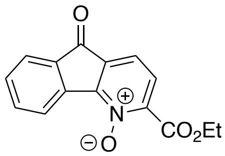

S1 ^{1}H
400 MHz
$CDCl_3$

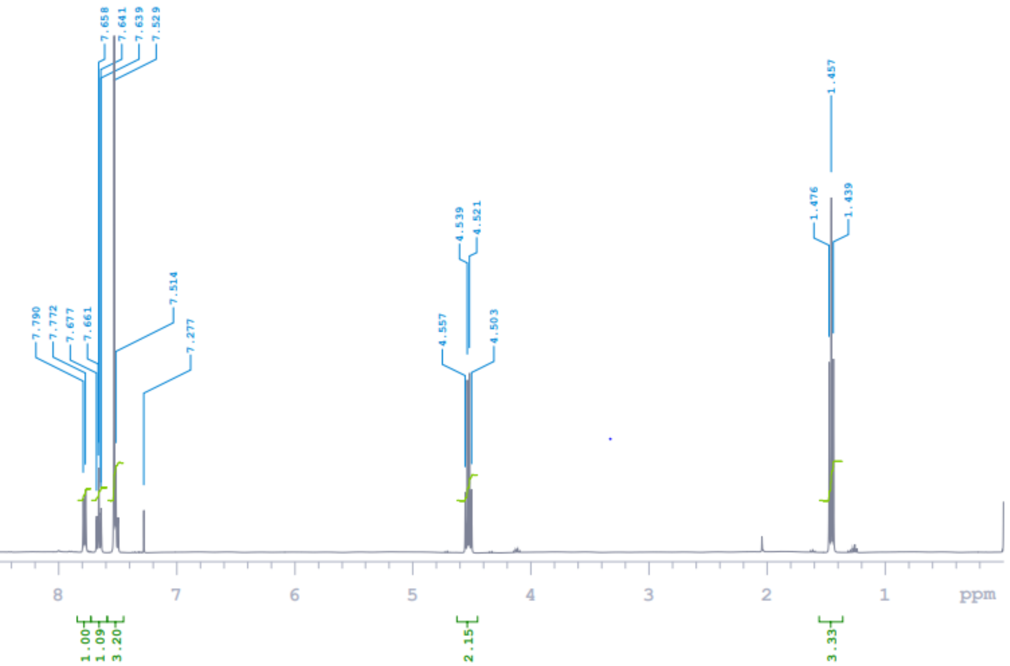

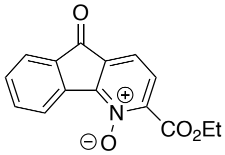

S1 $^{13}C\{^{1}H\}$
100 MHz
$CDCl_3$

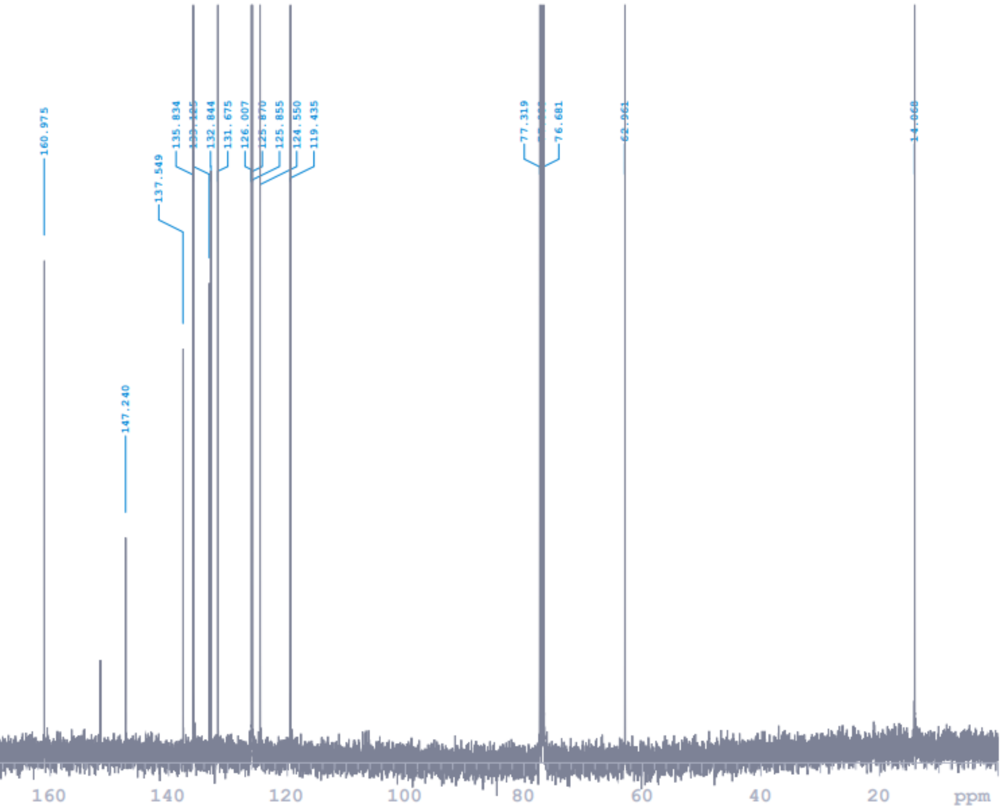

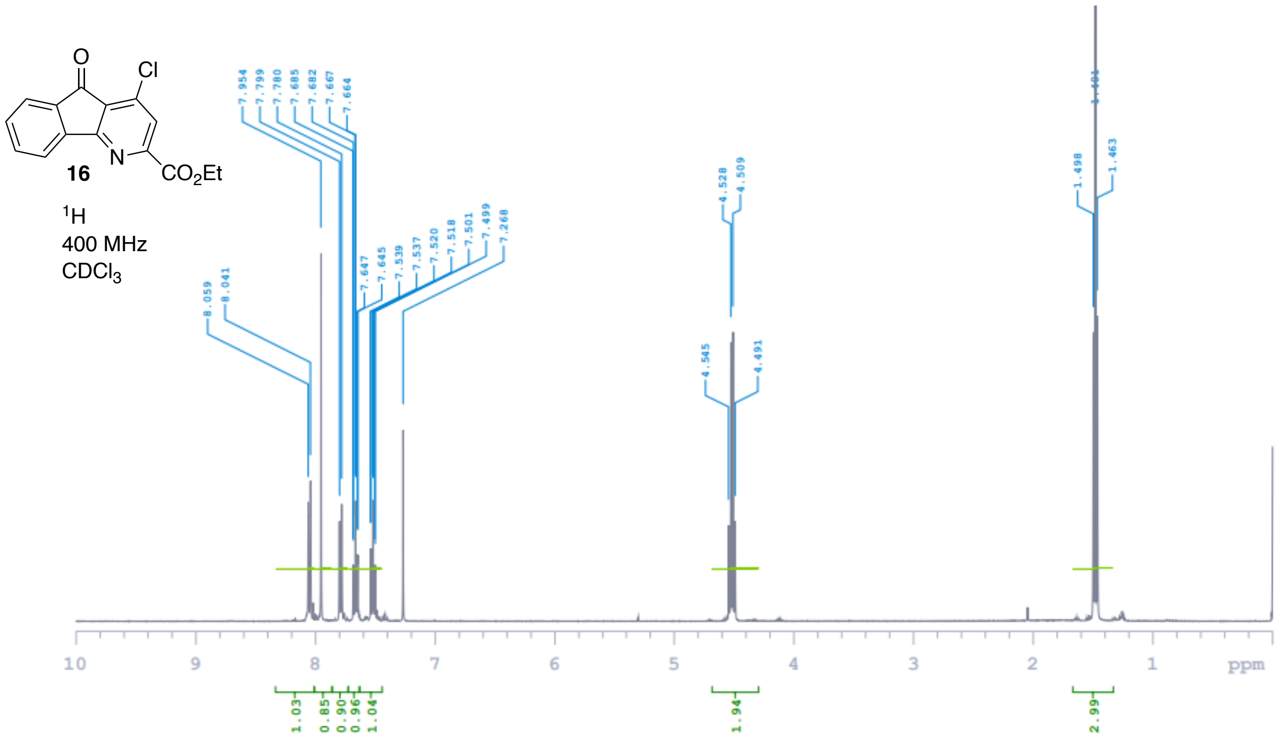

O
Cl
N
16
CO_2Et
^1H
400 MHz
$CDCl_3$
8.059
8.041
7.954
7.799
7.780
7.685
7.682
7.667
7.664
7.647
7.645
7.539
7.537
7.520
7.518
7.501
7.499
7.268
4.545
4.528
4.509
4.491
1.498
1.463
10
9
8
7
6
5
4
3
2
1
ppm
1.03
0.85
0.90
0.96
1.04
1.94
2.99

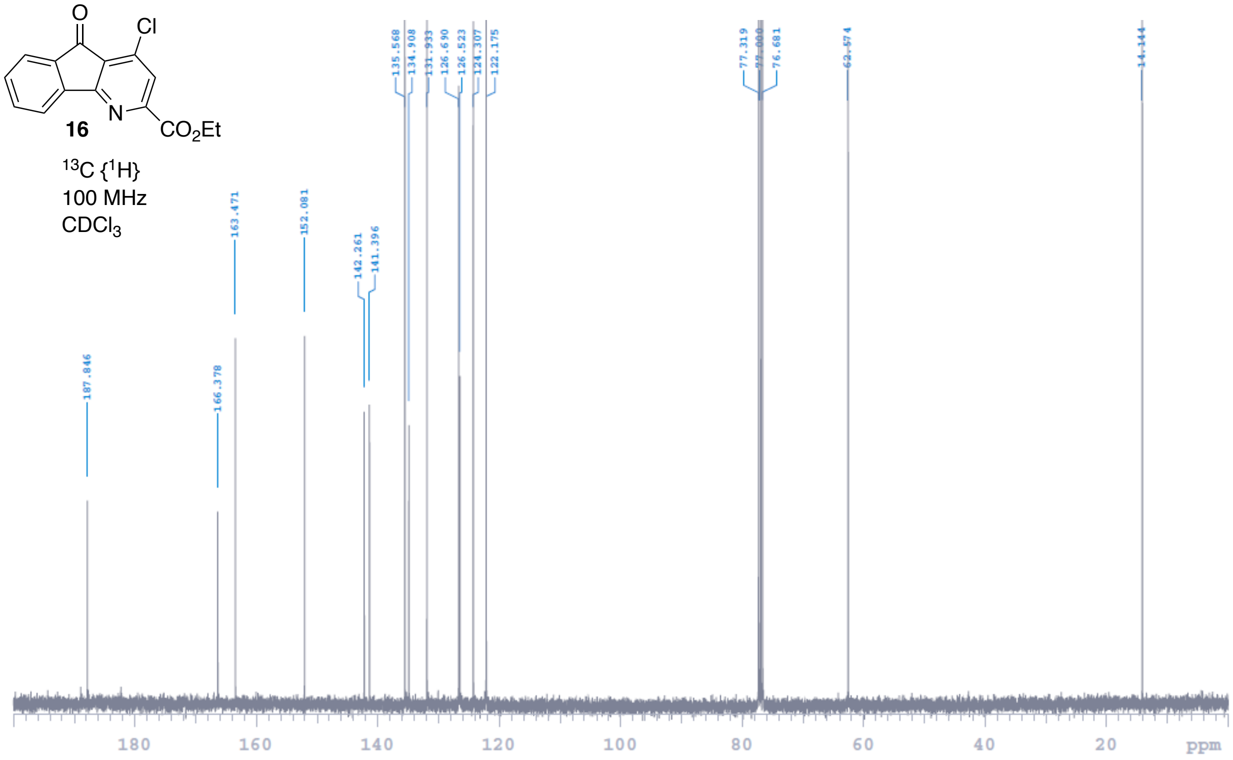

O
Cl
N
16
CO_2Et
$^{13}C\{^1H\}$
100 MHz
$CDCl_3$
187.846
166.378
163.471
152.081
142.261
141.396
135.568
134.908
131.933
126.690
126.523
124.307
122.175
77.319
76.681
14.144
180
160
140
120
100
80
60
40
20
ppm

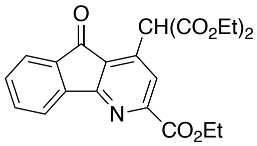

S2 ^{1}H
400 MHz
$CDCl_3$

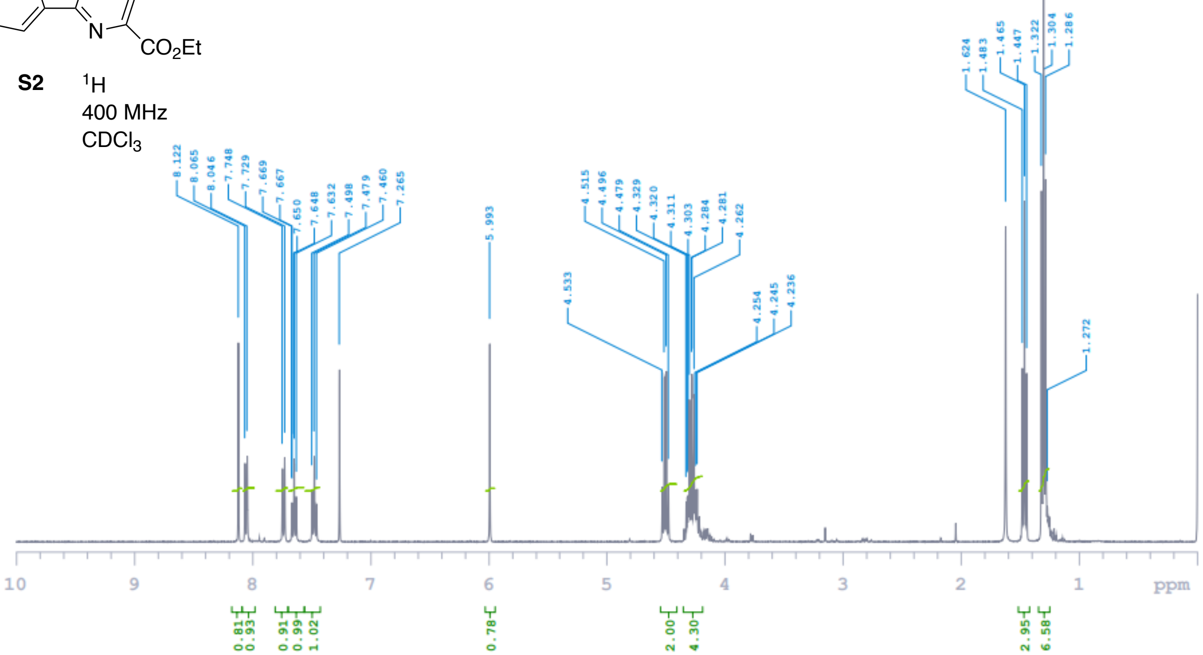

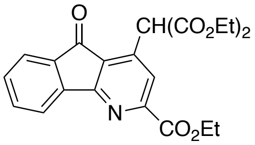

S2 $^{13}C\ \{^{1}H\}$
100 MHz
$CDCl_3$

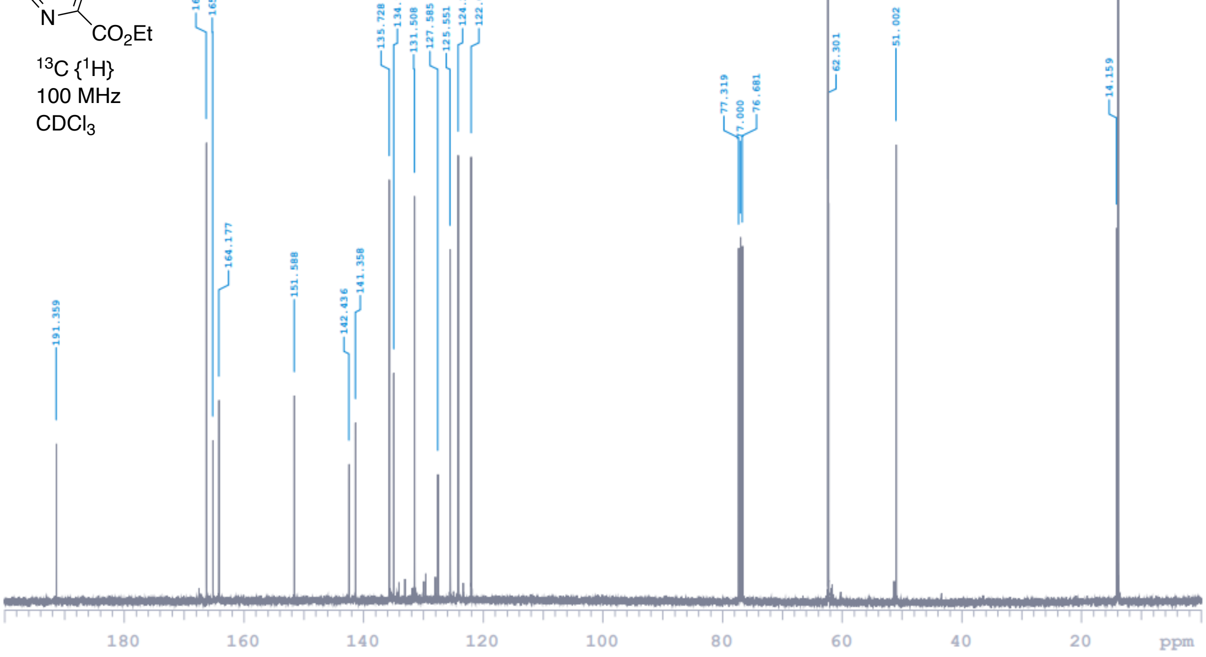

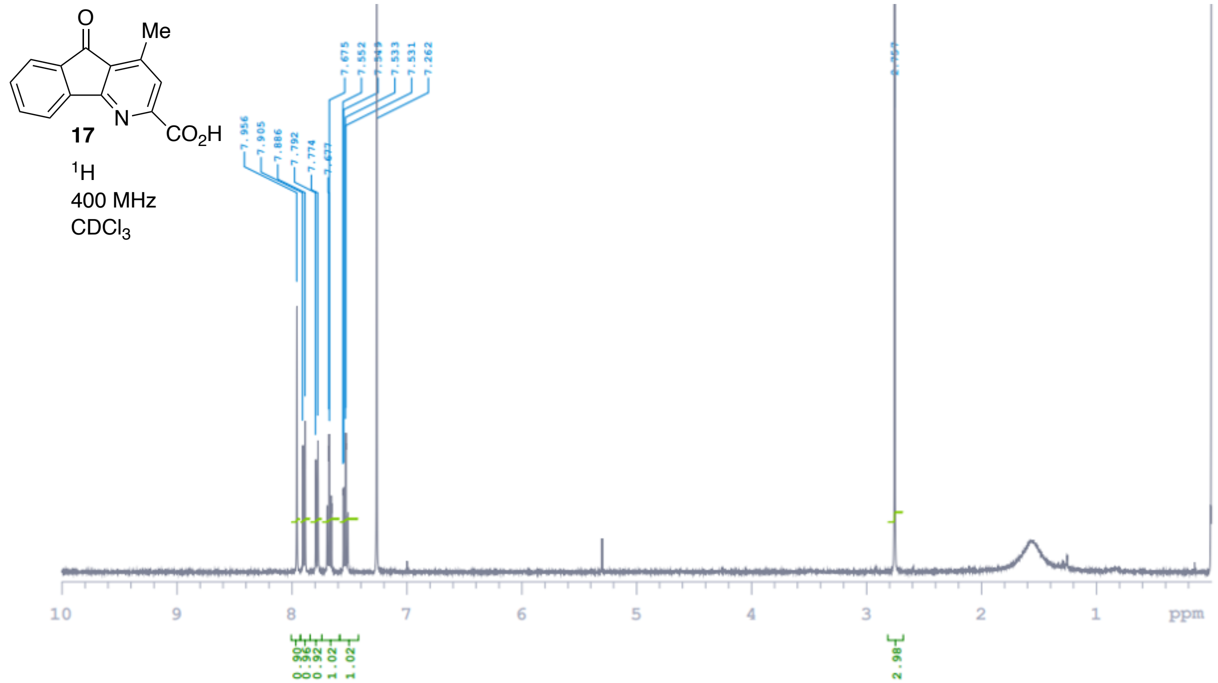
O
Me
N
CO2H
17
1H
400 MHz
CDCl3
10
9
8
7
6
5
4
3
2
1
ppm

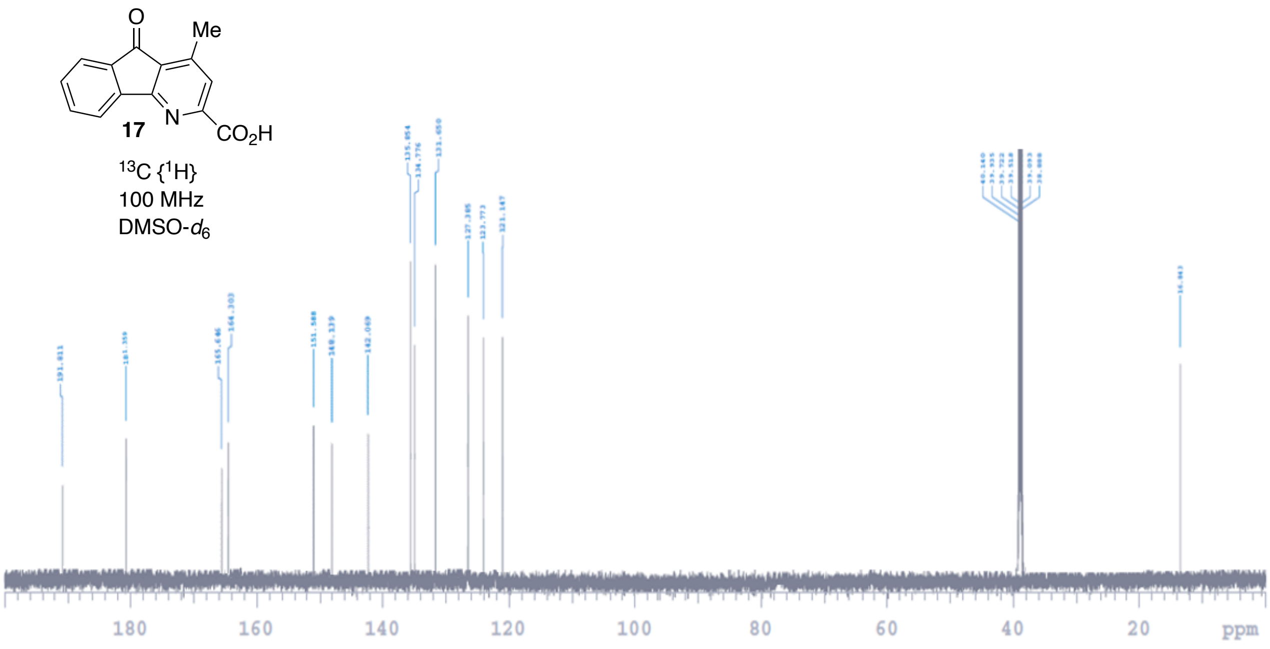
O
Me
N
CO2H
17
13C {1H}
100 MHz
DMSO-d6
180
160
140
120
100
80
60
40
20
ppm

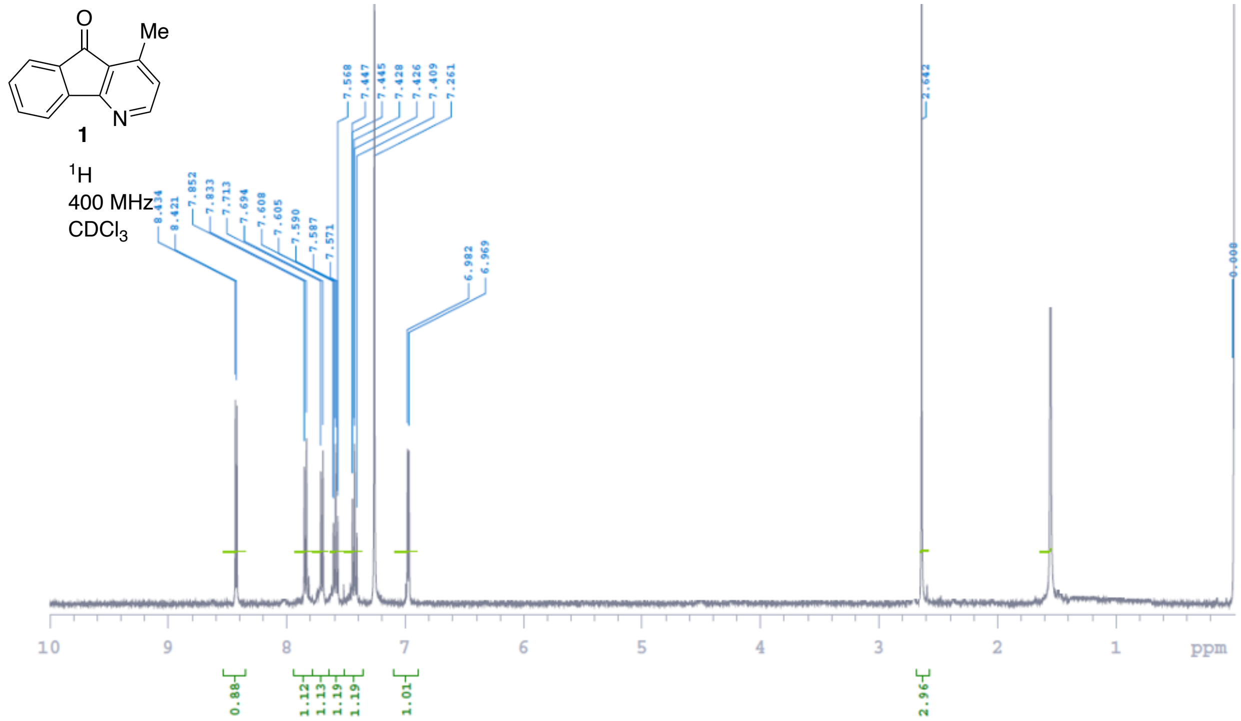
O
Me
N
1
¹H
400 MHz
CDCl3
8.434
8.421
7.852
7.833
7.713
7.694
7.608
7.605
7.590
7.587
7.571
7.568
7.447
7.445
7.428
7.426
7.409
7.261
6.982
6.969
2.642
0.000
10 9 8 7 6 5 4 3 2 1 ppm
0.88
1.12
1.13
1.19
1.19
1.01
2.96

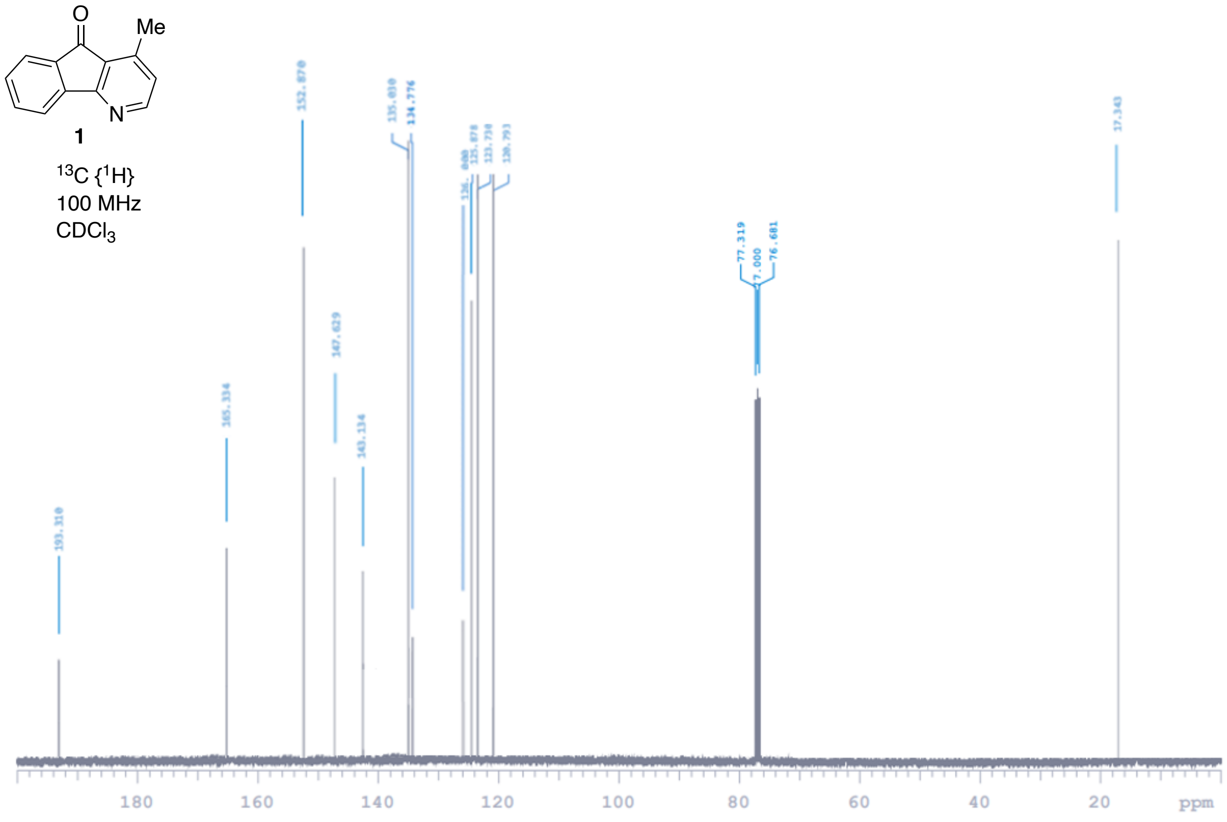
O
Me
N
1
¹³C {¹H}
100 MHz
CDCl3
193.310
165.334
152.870
147.629
143.134
135.030
134.776
126.000
125.878
123.738
120.793
77.319
77.000
76.681
17.343
180 160 140 120 100 80 60 40 20 ppm
